# High-resolution quantitative determination of dielectric function by using scattering scanning near-field optical microscopy

**DOI:** 10.1038/srep11876

**Published:** 2015-07-03

**Authors:** D. E. Tranca, S. G. Stanciu, R. Hristu, C. Stoichita, S. A. M. Tofail, G. A. Stanciu

**Affiliations:** 1 Center for Microscopy - Microanalysis and Information Processing, University Politehnica of Bucharest; 2 Department of Physics and Energy, University of Limerick.

## Abstract

A new method for high-resolution quantitative measurement of the dielectric function by using scattering scanning near-field optical microscopy (s-SNOM) is presented. The method is based on a calibration procedure that uses the s-SNOM oscillating dipole model of the probe-sample interaction and quantitative s-SNOM measurements. The nanoscale capabilities of the method have the potential to enable novel applications in various fields such as nano-electronics, nano-photonics, biology or medicine.

Scattering scanning near-field optical microscopy (s-SNOM)^1^,^2^,^3^,^4^,^5^ has attracted massive interest in the past couple of decades because of its capabilities for probing the optical properties of unlabeled samples at sub-diffraction resolutions. To date, s-SNOM has been successfully employed for multiple applications such as nano-imaging^3^,^6^,^7^, characterization of plasmonic structures^8^,^9^,^10^,^11^, near-field spectroscopy^4^,^5^,^12^,^13^,^14^, nano-chemical characterization^15^,^16^, or for the measurement of the dielectric function in the infrared domain^17^,^18^. In the present work, we propose a method based on s-SNOM that allows the measurement of the dielectric function of a material with nanoscale lateral resolution. Associated with database matching^19^,^20^ this method can conduct to the identification of investigated materials.

Many different techniques – like Quantitative Phase Microscopy^21^, Trans-Illumination Microscopy^22^, Optical Coherence Tomography^23^,^24^, Multiphoton Microscopy^25^, or Refracted Near-Field Technique^26^ – had been reported to date to be capable of measuring, mapping or profiling the dielectric function (the refractive index). Compared to these, the method that we propose provides three massive advantages: (a) sub-wavelength resolution; (b) the possibility to perform measurements on any solid material sample, irrespectively of its transparency and (c) the method is suitable not only for dielectrics, but also for metals and semiconductors.

Essentially different from a Plasmonic Force Microscope^27^ used for local dielectric response mapping (where measuring extremely low forces may be an issue), s-SNOM is typically built as an upgrade^28^ to an Atomic Force Microscope (AFM) allowing for simultaneous s-SNOM/AFM imaging. In this configuration, the metal-coated tip of a nano-probe is brought into the proximity of a sample and is driven into sinusoidal oscillations above the sample’s surface, with the frequency *f*_*o*_. An external laser source laterally illuminates the tip, while the sample is moved point-by-point in a raster scan process. One of the key problems related to s-SNOM imaging is background signal, which affects the detection of the near-field scattered light. Two combined methods are typically used to diminish the background light: *higher harmonic demodulation* (HHD)^29^ and *pseudoheterodyne detection* (PD)^30^. HHD takes advantage of the nonlinear dependence of the near-field scattered light intensity on the tip – sample distance. As the tip is oscillating with the frequency *f*_*o*_ above the sample, demodulation on a higher harmonic *nf*_*o*_ assures an important suppression of the background^29^. PD refers to an interferometric detection method in which the near-field scattered light interferes with a reference beam (which has the same wavelength). The phase of the reference beam is modulated by means of a vibrating mirror (with frequency *M* and amplitude *A*), which causes the appearance of two side-bands around each harmonic component of the HHD signal. It has been proven that the background light does not affect these side-bands^30^, which have components located at *nf*_*o*_ ± *mM* (with 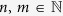). The amplitude of a spectral component *u*_*n,m*_ which is located at *nf*_*o*_ + *mM* is given by^30^:


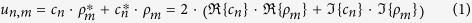


where *c*_*n*_ represents the Fourier spectral components of the near-field scattered light intensity (*σ*) and *ρ*_*m*_ are the spectral components of the reference beam intensity (*E*_*R*_). Symbols 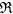 and 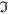 stand for the real and the imaginary parts, respectively.

The mathematical models that can accurately describe the physical phenomenon of the interaction between the incident beam, the probe (usually an AFM tip) and the sample have been thoroughly discussed to date^11^,^17^,^18^,^31^,^32^,^33^,^34^. The most popular models among these are the *Oscillating Point Dipole Model* (OPDM)^31^,^32^,^33^, which is based on the approximation of the probe with a sphere, and the *Finite Dipole Model* (FDM)^34^ in which the tip is treated as a conductive spheroid with physical characteristics of the probe. FDM has been successfully employed to date in the frame of various experiments based on infrared illumination for quantitative measurement of the dielectric function and the local absorption^17^,^18^; the results were obtained by employing Fourier Transform Infrared Spectroscopy (FTIR) and thus the applicability was limited to the infrared domain. The method that we propose combines the OPDM and s-SNOM imaging conducted in the visible domain in the purpose of quantitatively determining the dielectric function of an investigated material. While both FDM and OPDM based methods are capable of sub-wavelength resolution, the key advantage of using one over the other consists in reduced mathematical complexity for OPDM, as well as in the reduced number of parameters that are involved in this model. Probing the dielectric function at nanoscale holds massive potential for applications in various fields such as materials science^15^,^16^,^35^, nano-electronics^12^,^36^,^37^, biology^23^,^25^,^38^,^39^, or medicine^24^,^40^,^41^,^42^.

## Results

The proposed method for measuring the dielectric function is based on calculating a calibration factor between an experimental image and the OPDM-based simulated signal in the case of an investigated material of well-known dielectric function, under a particular s-SNOM imaging configuration. Once this calibration factor is known, it is further on used for determining the dielectric function of a second material present on the investigated sample, which is initially unknown. This can be achieved using the experimental s-SNOM image generated by the unknown material together with the calibration factor and running the OPDM backwards. Calculating the dielectric function of the second material allows for its exact identification via database matching^19^,^20^.

Before going further with the description of the implemented algorithm, two observations need to be emphasized.

O1: an important step of the algorithm consists in determining the value of a particular Fourier spectral component *c*_*n*_ corresponding to the investigated material with unknown dielectric function. The components *c*_*n*_ are functions of the local dielectric function of the sample, *c*_*n*_ = *f* (*ε*_*s*_) and are complex numbers; to determine both real and imaginary parts, one will need a set of two equations. This is the reason for which two images detected from two successive spectral components (located at *nf*_*o*_ + *mM* and *nf*_*o*_ + (*m* + 1)*M*) need to be used. All our calculations and experiments were done for *n* *=* *2* and *m* *=* *1*.

O2: the pixel value of an s-SNOM image *I*_*n,m*_ (achieved for the frequency *nf*_*o*_ + *mM*) is proportional with the amplitude of the harmonic component *u*_*n,m*_ on which the detection is employed^17^,^30^:


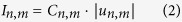


The parameter *C*_*n,m*_ plays the role of a calibration factor and it is specific to particular setup configurations consisting in detector sensitivity, the output scale of the lock-in amplifier, its amplifier factor, offsets, etc.

### Algorithm for determination of the dielectric function of an unknown material

Supposing a sample containing two different materials from which one of them is a material with known dielectric function, the algorithm for measuring the dielectric function of the other material is described in the following:

1. The first step consists in collecting two images, *I*_2,1_ and *I*_2,2_ detected at the frequencies 2*f*_*o*_ + *M* and 2*f*_*o*_ + 2*M*, respectively, while recording with sufficient precision the values of the configuration parameters involved (introduced in the Methods section). The image areas of the well-known material will be annotated as the sub-images 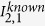 and 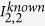. Similarly, the image areas of the unknown material will be annotated as the sub-images 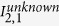 and 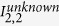.
2. The second step is to use the experimental values of the configuration parameters and the equations in the OPDM mathematical model to calculate the corresponding 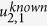 and 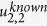 amplitudes for the material of known dielectric function.
3. The third step is to calculate the calibration factors from equation (2) and to determine the corresponding 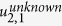 and 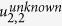 amplitudes for the material with unknown dielectric function.
4. The fourth step is to resolve the following set of equations based on the equation (1) in order to determine the real and imaginary parts of the spectral component 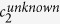 of the near-field scattered light signal for the unknown material:

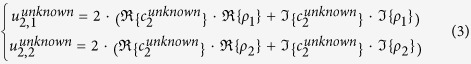
5. The fifth step consists in resolving the equation 
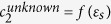
, thus obtaining the dielectric function of the unknown material. Knowing the dielectric function of a particular material allows for its facile identification via database matching^19^,^20^.

It can be observed that the entire algorithm is based on the proportional relationship between the results generated by the mathematical model and the experimental results. It is important to note that this relationship is valid as long as the values of the s-SNOM configuration parameters are well known, as these are needed for calculating the dielectric function based on the mathematical model that we introduced.

Demonstration of the method. For demonstrating the proposed method we use two samples, one that contains Si and SiO_2_ regions, and one that contains Pt and Al_2_O_3_ regions. Both samples are introduced in the Methods section. For each of these two samples, we run the experiment in two scenarios: in the first scenario we consider the first material as known, and the second material as the material of supposedly unknown dielectric function, and in the second scenario we switch roles. Results for the Si/SiO_2_ sample are relevant for samples containing semiconductor/dielectric materials, while the results for the Pt/Al_2_O_3_ sample are relevant for metallic/dielectric materials.

### Application of the algorithm on the Si/SiO2 sample

**Figure 1:**
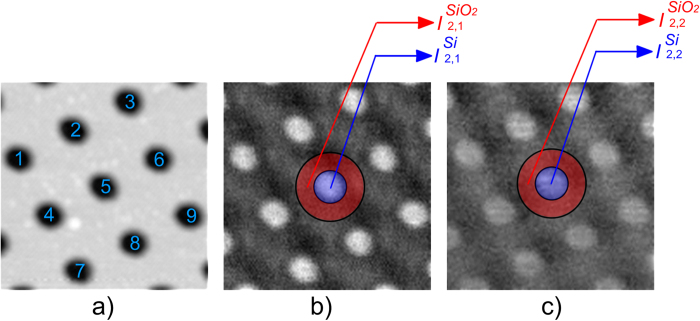
2 × 2 μm images of the Si/SiO_2_ sample; **a**) AFM image with Si regions indicated by numbers; **b**) s-SNOM image, detection on 2*f*_*o*_ + *M*; **c**) s-SNOM image, detection on 2*f*_*o*_ + 2*M*. In the s-SNOM images the central Si region is marked with blue and the surrounding SiO_2_ region is marked with red.

In the first experiment, SiO_2_ will be considered the unknown material whose dielectric function we want to determine for the used wavelength of 638 nm, while Si will be regarded as the material of known dielectric function. For sample areas containing both Si and SiO_2_ regions, two images *I*_2,1_ and *I*_2,2_ are collected at the frequencies 2*f*_*o*_ + *M* and 2*f*_*o*_ + 2*M*, respectively. In the AFM image illustrated as Fig. 1a, we mark nine Si regions. To demonstrate the proposed method, for each Si region the dielectric function of the corresponding SiO_2_ surrounding region is determined for the wavelength of 638 nm. In Fig. 1b,c the corresponding areas of the two materials are graphically delimited for the central region (region 5 in Fig. 1a). Average pixel values are calculated for each area to obtain 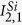, 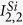, 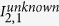 and 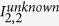 (as shown in Fig. 1). The area of each circle that delimits a Si region is chosen by considering all the pixels with values higher than 10% of the local intensity peak; the area of the second circle used for delimiting the SiO_2_ surrounding region is chosen so that the ratio between the Si and SiO_2_ areas equals the overall ratio between the areas of Si and SiO_2_ of the whole image.

Using the algorithm described in the previous sub-section, we calculate nine values of the dielectric function, one for each of the nine regions of supposedly unknown dielectric function (illustrated in Fig. 1). We calculated the average between the nine measured values and the mean absolute deviation (theoretical value*: ε*_*SiO2*_ *=* *2.379* for 638 nm):


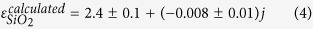


The second experiment ran on the Si/SiO_2_ sample demonstrates the capability of the method to determine the complex dielectric function of a semiconductor. In this second experiment the Si areas are considered of supposedly unknown dielectric function, while considering SiO_2_ as the known material. In this case, the average value, together with the mean absolute deviation for Si will be:


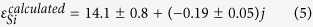


The theoretical value: *ε*_*Si*_ *=* *14.996-0.144j* for 638 nm.

### Application of the algorithm on the Pt/Al2O3 sample

**Figure 2:**
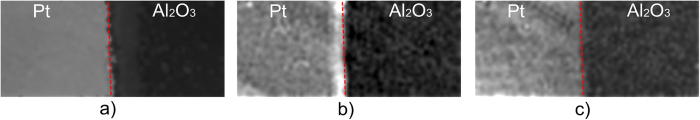
10 × 5 μm images of the Pt/Al_2_O_3_ sample; **a**) AFM image; **b**) s-SNOM image, detection on 2*f*_*o*_ + *M*; **c**) s-SNOM image, detection on 2*f*_*o*_ + 2*M*.

The method for dielectric function determination was applied on a second sample containing a Pt/Al_2_O_3_ boundary area (see Methods section and Fig. 2). Because of significant differences in the shape of the features that are present on the surface of different samples, the regions corresponding to certain materials cannot be defined always by specific geometric shapes as in the previous experiment. Thus, for this sample, instead of using circular and donut areas (as for the first sample), the measurements are done along horizontal scanning lines of the images, in the purpose of emphasizing the versatility of the presented method. For a single scanning line, the pixel value for Pt was obtained as an average of the pixels contained in the Pt part of the scanning line, and the pixel value for Al_2_O_3_ was obtained as an average of the pixels contained in the Al_2_O_3_ part of the scanning line.

The determinations were done in a similar manner with the ones performed for the Si/SiO_2_ sample. In a first scenario, Al_2_O_3_ was considered as a known material and the dielectric function for Pt was determined at the wavelength of 638 nm, and in a second scenario Pt was considered as the known material and the dielectric function for Al_2_O_3_ was determined at the same wavelength. Ten different scanning lines were used to calculate the average and the mean absolute deviation. For this sample, the method returned the following results for the dielectric function of Pt and Al_2_O_3_, respectively:


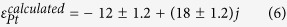



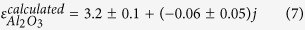


Theoretical values are *ε*_*Pt*_ *=* *–11.834–19.773j*, and *ε*_*Al2O3*_ *=* *3.118* (for 638 nm).

## Discussion

The obtained results demonstrate that the dielectric function of a material can be measured with good precision using the proposed method that combines s-SNOM imaging and the OPDM. The key requirement of this method is that in the s-SNOM image used as support for measuring the dielectric function of one or more materials, s-SNOM data collected on one or more materials of known dielectric function needs to be included as well. Using the s-SNOM data collected in the regions corresponding to the material of known dielectric function, a calibration factor is calculated and used for determining the dielectric function of other materials contained in the same s-SNOM image.

Since the resolution of an s-SNOM setup depends on the radius of curvature of the metallic probe used for scanning the sample, typically lying in the range of 10-40 nm^27^,^43^,^44^,^45^, this method allows for determining the dielectric function of nanoscale sample components, which enables new perspectives for novel characterization methods of high potential usefulness for fields such as material science, nano-electronics, biology, medicine or others.

The proposed method exhibits versatility with respect to the approaches used for defining the regions corresponding to different materials. Thus, among the most important factors is the exact localization of the known material. Afterwards, one can use the pixels contained in areas of different shapes (as in the first experiment), or even the pixels contained along a scanning line of the images (like in the second experiment).

The performed experiments demonstrate that the proposed method for high-resolution dielectric function measurement is highly effective for different material classes such as dielectrics, semiconductors and metals. Comparing the averaged measured values (see equations (4, 5, 6, 7)) with the actual values of the dielectric functions for the four materials involved in the experiments (see Methods), small discrepancies can be observed and they are mainly connected to measurement errors.

As the resolution of s-SNOM images is not limited by optical diffraction, a high impact of these quantitative measurements is expected – especially in the field of electronic nanochips industry or in the constantly-growing field of photonic integrated circuits. For example, in the actual requirements in MOSFETs industry, the optical constant measurement of SiO_2_ thin films stands particularly important^36^. On the other hand, the AFM has become indispensable in the semiconductor industry for dimension metrology^46^.

The main limitation of this method is given by the dielectric function dependency on the light wavelength, as our method offers the possibility to measure the corresponding dielectric function for a single wavelength at one time.

In summary, a new method for quantitative high-resolution measurement of the dielectric function was introduced. The performed experiments demonstrate high measurement precision and enforce the idea that a combined s-SNOM/AFM system can be regarded as a powerful tool for simultaneous metrology and optical properties measurements. Such a tool has the potential to enable novel applications in the fields of nano-electronics, nano-photonics, material science, biology or medicine.

## Methods

### Materials

The semiconductor/dielectric sample that we have used consists in a SiO_2_ thin layer (26.6 nm thick) deposited on a Si substrate. The sample contains periodic circular holes, with a diameter of 500 nm, that penetrate the SiO_2_ layer reaching the Si substrate. The values for the dielectric functions of the two materials are as follows: *ε*_Si_ = *14.996–0.144j* for Si, and *ε*_SiO2_ = *2.379* for SiO_2_ (at wavelength 638 nm)^19^,^20^.

The metal/dielectric sample was a 10 nm thick rectangular domain of Pt deposited on an Al_2_O_3_ substrate and the surface boundary between the two materials was investigated. The values of the dielectric functions of the two materials are as follows: *ε*_Pt_ = *−11.834–19.773j* for Pt, and *ε*_Al2O3_ = *3.118* for Al_2_O_3_ (at wavelength 638 nm)^19^,^20^.

The values of the dielectric functions at 638 nm were obtained by employing the least squares method to the data provided by the available databases^19^,^20^.

### Mathematical model and calculations

Previous studies have already shown that the intensity of the near-field scattered light *σ* is proportional with the amplitude of the incident light phasor *E*_*o*_ and the effective polarizability, *α*_*eff*_ , where the effective polarizability has the form^31^,^32^,^33^:


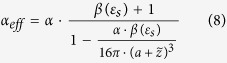


In equation (8), α stands for the polarizability of the tip, with its formula:


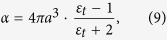


where *a* is the tip diameter and *ε*_*t*_ is the electric permittivity of the tip. The reflection coefficient *β*(*ε*_*s*_) is a parameter that depends on the local dielectric function of the sample *ε*_*s*_ by the relation:


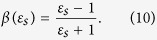



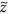
 is the instantaneous distance from the tip of the probe to the sample’s surface and it can be defined as:


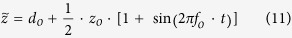


Here, *d*_*o*_ stands for the minimum separation distance between the tip and the sample during the probe oscillation above the sample, *z*_*o*_ is the oscillation amplitude of the probe, *f*_*o*_ is the oscillation frequency of the probe and *t* is time.

Based on the equations presented until now, the intensity of the near-field scattered light (as a function of *β* and time) can be rewritten in the following form:


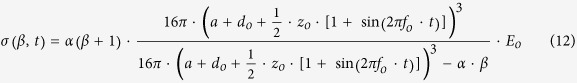


Starting from this point, one can spectrally analyze the function given by equation (12) using the exponential Fourier transformation method. Using the variable-changing *u*(*t*) = 2*πf*_*o*_·*t*, the Fourier coefficients *c*_*n*_ will be given by:


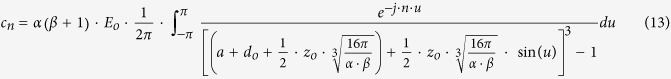


**Figure 3:**
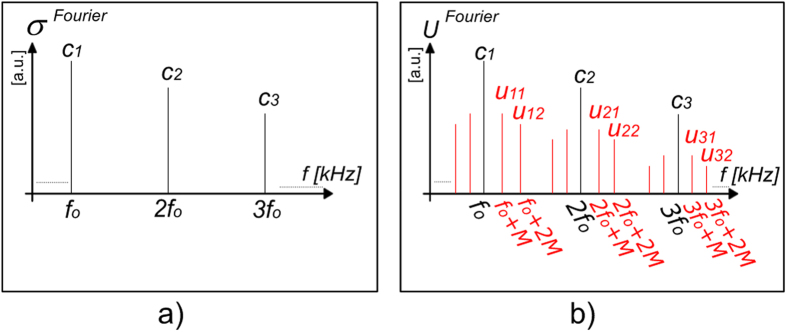
Fourier spectra of the detected signal; **a**) The frequency spectrum of the near-field scattered light modulated by the vibration of the nano-probe with the frequency *f*_*o*_; **b**) The frequency spectrum of the pseudo-heterodyne signal *U*, which is the result of the interference between the amplitude-modulated near-field light and the phase-modulated reference beam.

In a brief evaluation of equation (13), it can be observed that the only variable implied is *β,* which is linked to the sample’s electric permittivity *ε*_*s*_ by equation (10) thus, the notation *c*_*n*_ = *f*(*ε*_*s*_) is justified. The other parameters are usually known because they characterize the system setup. Fig. 3a illustrates the schematic frequency spectrum of the near-field scattered light, *σ*.

The integral in equation (13) is not a common one and its calculation requires special mathematical algorithms; calculating its expression for a general variable *n* can be regarded as difficult. However, for a given value for *n*, the integration complexity decreases, allowing for calculation via software computational engines.

In the pseudo-heterodyne scheme, the near-field scattered light interferes with the reference beam *E*_*R*_, which can be mathematically written as:


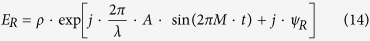


where *ρ* is the amplitude of the reference beam phasor, *A* is the oscillation amplitude of the reference mirror, *λ* is the wavelength of the beam, *M* is the oscillation frequency of the reference mirror, *Ψ*_*R*_ is the mean phase difference between the two interferometric pathways and *t* is time.

The mathematical function in equation (14) can be expanded in a Fourier series^47^:


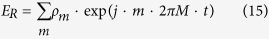


where the coefficients *ρ*_*m*_ are the Fourier coefficients given by:
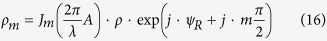


In equation (16), *J*_*m*_ stands for the Bessel function of order *m*.

The interference signal *U* between the near-field scattered light *σ* and the reference beam *E*_*R*_ will have the spectral components *u*_*n,m*_, introduced by equation (1).

In Fig. 3b we represent the frequency spectrum of the interference signal *U* with the side-bands around each harmonic component of the cantilever oscillation frequency^30^.

### Software calculations and simulations

Calculations and simulations have been performed using the WOLFRAM|Alpha online platform and the MATLAB software platform. More precisely, the WOLFRAM|Alpha online platform was used for calculating the integral functions required by the spectral components analysis of the s-SNOM signal, while the MATLAB software platform was used for s-SNOM signal simulation, image analysis and calculation of the calibration factors.

### Experimental data acquisition

Experimental data was collected by using a homemade pseudo-heterodyne s-SNOM setup upgrading an AFM Quesant 350^28^. The s-SNOM configuration parameters during data acquisition were set to the following values: beam wavelength, *λ* = 638 nm; oscillation frequency of the probe, *f*_*o*_ = 60 kHz; oscillation amplitude of the probe, *z*_*o*_ = 50 nm; oscillation frequency of the reference mirror, *M* = 1000 Hz; oscillation amplitude of the reference mirror, *A* = 267 nm; mean phase difference between the two interferometric pathways (in the pseudo-heterodyne scheme), *Ψ*_*R*_ = π; ratio between the reference and the incident beam intensities (*ρ*_*o*_ and *E*_*o*_, respectively) is 1. The investigating Pt-coated nano-probe has a tip radius of curvature less than 35 nm. The values of the dielectric function of Platinum is ε_Pt_ = *−11.834–19.773j* at the wavelength of 638 nm^19^,^20^.

## Additional Information

**How to cite this article**: Tranca, D. E. *et al.* High-resolution quantitative determination of dielectric function by using scattering scanning near-field optical microscopy. *Sci. Rep.* **5**, 11876; doi: 10.1038/srep11876 (2015).
